# The Role of RNA Methyltransferase *METTL3* in Normal and Malignant Hematopoiesis

**DOI:** 10.3389/fonc.2022.873903

**Published:** 2022-04-28

**Authors:** Xia Wu, Wu Ye, Yuping Gong

**Affiliations:** Department of Hematology, West China Hospital, Sichuan University, Chengdu, China

**Keywords:** *METTL3*, N6-methyladenosine, normal hematopoiesis, malignant hematopoiesis, inhibitor

## Abstract

m^6^A modification is the most common modification in eukaryotes. *METTL3*, as a core methyltransferase of m^6^A modification, plays a vital role in normal and malignant hematopoiesis. Recent studies have shown that *METTL3* is required for normal and symmetric differentiation of hematopoietic stem/progenitor cells (HSPCs). Moreover, *METTL3* strongly impacts the process and development of hematological neoplasms, including the differentiation, apoptosis, proliferation, chemoresistance, and risk of tumors. Novel inhibitors of *METTL3* have been identified and studied in acute myeloid leukemia (AML) cells. STM2457, a selective inhibitor of *METTL3*, has been identified to block proliferation and promote differentiation and apoptosis of AML cells without impacting normal hematopoiesis. Therefore, in our present review, we focus on the structure of *METTL3*, the role of *METTL3* in both normal and malignant hematopoiesis, and the potential of *METTL3* for treating hematological neoplasms.

## Introduction

Epigenetic modifications have been identified to be involved in many physiological and pathological processes in most eukaryotes without DNA sequence changes (1), including DNA methylation, histone modification, RNA methylation, and noncoding RNA regulation (2). Unlike DNA methylation and histone modification, RNA methylation is still at an infant stage. Among RNA methylation modifications, N6-methyladenosine (m^6^A) is the most abundant internal modification of messenger RNA (mRNA) (3), which was first discovered in Novikoff hepatoma cells in 1974 (4). However, due to the lack of robust methods to detect the precise modification sites of m^6^A in mRNA, interest in m^6^A research has been hindered significantly. It was not until 2011 that fat mass and obesity-associated protein (*FTO*) was discovered as a m^6^A demethylase, indicating the reversibility of the m^6^A modification on mRNA (5). Meanwhile, detection technology has been largely improved and has benefited the investigation of m^6^A modification on mRNA. Dominissini et al. and Meyer et al. independently used high-throughput sequencing to detect m^6^A modification at the whole transcriptome level, revealing the main distribution of m^6^A near stop codons, 3′ or 5′-untranslated terminal regions (UTRs), and long exons (5, 6). Due to the two critical advances, the enthusiasm and motivation of m^6^A research have been refueled, resulting in a flood of studies on the m^6^A modification on mRNA in eukaryotes.

### m^6^A Methylation Composition

**Figure 1:**
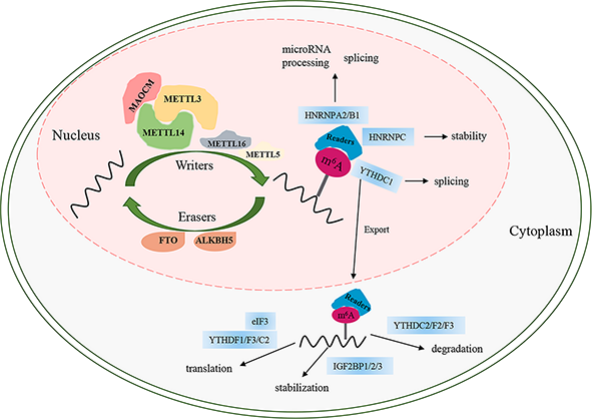
The process of m^6^A modification. m^6^A RNA methylation is regulated by “writers”, “erasers”, and “readers”. MAOCM, m^6^A-METTL-associated complex, composed of WTAP, VIRMA, RBM15/15B, and ZC3H13.

Similar to DNA methylation, m^6^A is a reversible and dynamic process regulated by three categories of enzymes, namely, “writers,” “erasers,” and “readers” (**Figure 1**). At present, *FTO* and ALKB homolog 5 (*ALKBH5*) are the only two identified “erasers” that are responsible for reversing m^6^A (5, 7) (**Figure 1**). *FTO*, the first m^6^A demethylase identified in 2011, has strongly promoted the development of research on m^6^A. The demethylation activity of ALKBH5 significantly impacts mRNA export, RNA metabolism, and mRNA processing factor assembly (8). The final biological function of m^6^A is mainly associated with m^6^A “readers” that recognize sites of m^6^A and induce it to bind to the target sites to perform different functions involving mRNA degradation, translation, splicing, stability, and export (9–15) (**Figure 1**). m^6^A can be recognized by a set of RNA-binding proteins, including YT521-B homology (YTH) domain family proteins (*YTHDF1/2/3*, *YTHDC1/2*) (10–13, 16), insulin-like growth factor 2 mRNA-binding proteins (IGF2BPs, including IGF2BP1/2/3) (15), heterogeneous nuclear ribonucleoproteins (including *HNRNPA2B1*, *HNRNPG*, and *HNRNPC*), and eukaryotic translation initiation factor 3 (eIF3) (9, 17, 18). m^6^A is installed by methyltransferases (writers), which comprise several different “writer” proteins, including methyltransferase-like 3/5/14/16 (*METTL3*/5/14/16), Wilms tumor 1-associated protein (*WTAP*), Vir-like m^6^A methyltransferase associated (VIRMA, also called KIAA1429), RNA binding motif protein 15/15B (RBM15/15B), zinc finger CCHC-type containing 4 (ZCCHC4), and zinc finger CCCH-type containing 13 (*ZC3H13*) (19) (**Figure 1**). Among the methyltransferases, *METTL3* is the only one that has the S-adenosyl methionine (SAM)-binding protein in the catalytic pocket and composes a stable methyltransferase complex (MTC) heterodimer with *METTL14* at 1:1 to exert methylation activity (20). Moreover, the activity of the *METTL3*/*METTL14* core complex is assisted by an additional regulatory complex (known as MACOM, a m^6^A-METTL-associated complex) composed of *WTAP*, *VIRMA*, *RBM15/15B*, and *ZC3H13*. *WTAP* contributes to the heterodimer being located in nuclear speckles to complete m^6^A modification (21, 22). *VIRMA* recruits the catalytic core complex *METTL3*/*METTL14*/*WTAP* in the 3′UTR and is near the stop codon to methylate (23). RBM15/15B, as an X-inactive specific transcript-binding protein (XIST-binding protein), is associated with XIST-mediated gene silencing and regulates the m^6^A modification in XIST (24). *ZC3H13* interacts with the m^6^A machinery and contributes the MTC to the mRNA-specific sites by binding factor Nito (25). *ZCCHC4* acts on 28S rRNA by m^6^A modification and impacts mRNA translation (26, 27). *METTL5* is the 18S rRNA m^6^A methyltransferase (26). *MTTL16*, a novel methyltransferase, is responsible for modifying the m^6^A modification of A43 in U6 small nuclear RNA and catalyzing m^6^A within a hairpin in MAT2A (28) (**Figure 1**).

Because *METTL3* plays a critical role in catalytic activity, many studies regarding its biological function in cancers have been widely reported, including lymphoma (29, 30), leukemia (31–33), breast cancer progression (34), liver cancer (35), glioblastoma (36, 37), bladder cancer (38, 39), gastric cancer (40), and lung cancer (41, 42). However, studies on m^6^A modification in hematology have not been systematically summarized thus far. Therefore, we will mainly focus on the role of *METTL3* in normal hematopoiesis and hematological neoplasms in the present review and discuss directions for future research and potential clinical applications of *METTL3* in hematological diseases.

### METTL3 Is a Core Protein for m^6^A Modification

**Figure 2:**
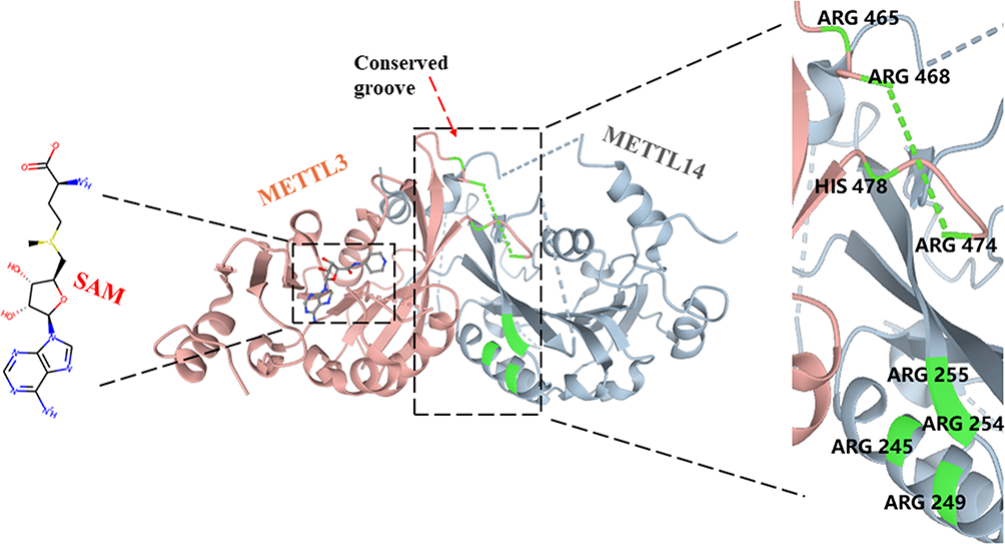
Structure of the METTL3-METLL14 complex.

*METTL3* (also known as MT-A70), a 70-kDa protein, was first identified as a m^6^A “writer” and is highly conserved in eukaryotes from yeast to human (43). Although *METTL3* forms a stable 1:1 heterodimer structure with METTL14 to exert higher methylation activity, it has been identified as the core catalytic enzyme of m^6^A methylation, and METTL14 mainly plays a role in the structure of MTC stabilization and recognizes target RNAs (20, 24, 44, 45). In contrast to METTL14, *METTL3* contains S-adenosyl methionine (SAM)-binding protein and its product S-adenosyl homocysteine (SAH) in the catalytic pocket, which were not observed in METTL14 (20, 44–46) (**Figure 2**). In addition, the catalytic site of *METTL3* contains a more conserved DPPW motif involved in coordinating the adenine of the acceptor substrate, while METTL14 has a more divergent EPPL sequence (20, 46, 47). After replacing the DPPW motif with APPW(D395) in *METTL3*, the methylation of the *METTL3*–METLL14 complex was significantly destroyed but was very lightly affected after changing EPPL to APPL in METTL14 (44). Moreover, due to the collision between the adenine moiety and the side chain (Trp211 and Pro362) residues in METTL14, the binding of SAM would be prevented in the METTL14 catalytic site (46, 48). Furthermore, *METTL3* also contains two CYS-CYS-HIS (CCCH)-type zinc binding motifs, which are critical for RNA methylation *in vitro* (44, 49). The methyltransferase domain of *METTL3* (MTD3) presents a classic α–β–α fold, including a mixed eight-strand β-sheet, four α-helices, and three 310 helices, which makes a special catalytic cavity for *METTL3*, while the catalytic site of METTL14 is relatively occluded (20, 44). The methyltransferase domain of METTL14 (MTD14) contains residues 165–378, which is near the N-terminal α-helical motif (NHM, residues 116–163) and the C-terminal motif (CTM, residues 380–402) (20). MTD3 mainly contains residues 369–570, making three loops to fence the *METTL3* catalytic cavity: gate loop 1 (residues 396–410), gate loop 2 (residues 507–515), and interface loop (residues 462–479) (20, 44). The two gate loops are adjacent to the SAM binding site and are associated with adenosine recognition, and the interface loop with the longer sequence allows *METTL3* and METTL14 to bind each other tightly (20, 44, 50). Meanwhile, 11 residues of *METTL3* are involved in SAM coordination, including D377, I378, Q550, N549, R536, D395, K513, H538, N539, E532, and L533 (20). Wang et al. further found that mutations of these residues completely abrogated methyltransferase activity (D377A, D395A, N539A, and E532A) or moderately weakened enzyme activity (R536, H538, N549, or Q550), while corresponding mutations of METTL14 have little effect on catalytic activity (20, 44). Between the interface of *METTL3* and METTL14, a highly conserved groove comprises Arg465, Arg468, His474, and His478 of *METTL3* and Arg245, Arg249, Arg254, and Arg255 of METTL14, which contributes to internal RNA binding (20, 46).

Furthermore, *METTL3* has also been reported to promote translation independently of its methyltransferase activity or downstream m^6^A reader proteins (51, 52). The function of *METTL3* in the cytoplasm promoting translation is to recruit the initiation factor eIF3 h to the translation initiation complex (51, 52). It can enhance epidermal growth factor receptor (EGFR) and TAZ protein expression independent of YTHDF1.

## The Biological Function of *METTL3*

More recent studies on *METTL3* in hematology have been reported, including the function of *METTL3* in normal and malignant hematopoiesis. *METTL3* has been discovered to be associated with differentiation, growth, and apoptosis in both normal and malignant cells. Moreover, it has also been revealed to impact chemoresistance in chronic myeloid leukemia (CML) and acute myeloid leukemia (AML), which would be a novel potential target molecule in hematological neoplasms. Therefore, we summarized the function of *METTL3* in normal hematopoiesis and several hematological malignancies (**Table 1**).

**Table 1:** Role of *METTL3* in normal and malignant hematopoiesis.

Type	Subjects	Target genes	Biology functions	Mechanism	Refs
Normal Hematopoiesis	Zebrafish	notch1a	Promotes HSPC generation and differentiation.	Downregulate notch1a expression; Inhibit endothelial Notch signaling activity	(14)
	Mouse	notch1a	Promotes HSPC generation through EHT. Promotes colony formation ability of HSPC	Facilitates the m6A methylation on Notch1 mRNA to inhibit endothelial Notch signaling activity	(53)
	BMC Mouse	*Myc*	Promotes HSCs differentiation Promotes HSCs colony formation ability Promotes cell-intrinsic HSC reconstitution *in vivo*	Reduce *Myc* mRNA translation	(54)
	Mouse	Myc	Maintains HSCs symmetrically differentiate Regulates HSCs number and function	Control Myc abundance Enhance Myc mRNA stability	(55)
	Mouse	Nr4a2, Bmi-1, p21, Prdm16	Maintains HSCs quiescence Regulates HSCs self-renewal	Regulating HSCs self-renewal genes Nr4a2, p21, Bmi-1, and Prdm16	(56)
	h-CBD	/	Inhibit myeloid differentiation, not affects apoptosis in HSPCs Promote cell proliferation and CFU	No study	(31)
	HELs	GYPA, HBA1, SPTB, EPOR, ALAS2	Maintains CD235a/GYPA expression in HEL cells Promote normal erythropoiesis in HSPCs	Regulates genes associating with erythropoiesis, such as GATA1, GATA2, KLF1, RUNX1, and SPI1 mRNAs	(57)
AML	AML cells; mouse	c-MYC, BCL2 ,PTEN	Inhibit differentiation and apoptosis of AML cells Promote proliferation	Increases c-MYC, BCL2, PTEN translation Blocks p-AKT pathway	(31)
	AML cells	SP1	Inhibits differentiation and promotes proliferation	Promotes SP1 mRNA translation	(32)
	AML cells	MYC	Inhibits differentiation and promotes proliferation	Promotes MYC mRNA translation	
	MSC	PI3K/AKT	Inhibits MSC adipogenesis and AML chemoresistance	Inhibits AKT1 translation Activate PI3K/AKT signaling pathway	(58)
AML	AML cells	p53; MDM2	Inhibits differentiation Promotes proliferation and cell cycle	Increase MDM2 stability and translation Inhibits p53 pathway	(59)
CML	K562	MYC; PES1	An oncogene in CML Promotes growth and viability of CML cells, including K562 cells and K562r	Promotes PES1 translation	(60)
	K562; KCL22; mouse	PTEN	Promotes chemoresistance and inhibits autophagy	Suppress PTEN stability by interacting with LINC00470	(61)
	K562, KCL22, MEG01, and BV173 cell	NEAT1	Enhances viability, and inhibit apoptosis	Inhibits NEAT1 degradation Promotes NEAT1 mRNA stability	(62)
ALL	Patients	no study	Lower expression in children with ETV6-RUNX1-positive ALL and relapse patients	No study	(63)
	Patients	no study	Three polymorphisms (rs1263801 C>G; rs1139130 A>G; rs1061027 A>C) of *METTL3* increase the risk of the common B type and MLL fusion type ALL in Southern Chinese children	No study	(33)
Lymphoma	Tissues and cells Mouse	PEDF	Promotes proliferation and viability	Promotes PEDF translation and activates Wnt/β-actine signaling	(29)

HSPC, hematopoiesis stem/progenitor cells; BMC, bone marrow cells; HSCs, hematopoiesis stem cells; EHT, endothelial-to-hematopoietic transition; h-UBD, human cord blood derived; HELs, human erythroid leukemia cells; CFU, colony-forming units; MSCs, mesenchymal stem cells; K562r, TKI imatinib mesylate-resistant K562 cell line.

### Normal Hematopoiesis

Normal hematopoiesis plays a vital role in hematology, presenting multipotent hematopoietic stem cells (HSCs) being differentiated into various mature cells of the blood system. The process is complex and multistep and is regulated by many factors, such as the transcription factor PU.1 for myeloid cells and C/EBPα for granulocyte/macrophage progenitors.

The first populations of hematopoietic stem cells (HSCs) are mainly produced by hemogenic endothelial cells (ECs), which later acquire a cell morphology and gene expression consistent with hematopoietic identity in a process called endothelial-to-hematopoietic transition (EHT) (64). In zebrafish, m^6^A has been reported to determine cell fate when EHT progresses to the earliest hematopoietic stem/progenitor cells (HSPCs) during embryogenesis (14). *METTL3* was found to be enriched in sorted endothelial cells and hemogenic endothelium, correspondingly affecting hematopoiesis (14). The deletion of *METTL3* results in impaired HSPC differentiation by activating Notch1 signaling. Increasing Notch signaling can abrogate the generation of hematopoietic cells by maintaining endothelial identity in EHT (14, 64). *Notch1a* m^6^A enrichment is significantly decreased in *METTL3* morphants, but the expression of *Notch1a* is increased in endothelial cells, resulting in a decrease in HSPC generation (14). Conversely, overexpression of *METTL3* inhibiting Notch1 activity could rescue this phenomenon in zebrafish (14). Additionally, the same phenotype was also observed in mice with *METTL3* knockdown. In 2018, this team reported that *METTL3* promotes HSPC generation by inhibiting Notch1 signaling in endothelial cells of the *Vec-Cre*; *METTL3^fl/fl^* mouse aorta-gonad-mesonephros (AGM) region, consistent with the phenomenon in zebrafish (53). In 2019, Heather Lee et al. performed a study on *Mx1-cre; METTL3^fl/fl^* mice and discovered that *METTL3* deletion has little impact on HSC self-renewal and quiescence but significantly affects HSC differentiation (54). The deletion of *METTL3* has resulted in a blocking of HSCs and an accumulation of HSCs by reducing *Myc* mRNA translation (54). Deleting *METTL3* in myeloid cells from *Lysm-cre; METTL3^fl/fl^* mice, they found that *METTL3* is not indispensable for mature myeloid cell maintenance or function (54). *Via Mx1-cre; METTL3^fl/fl^* mice, Cheng et al. reported that *METTL3* depletion in normal murine HSCs results in a decrease in Myc mRNA and protein levels. Furthermore, *METTL3* is required for normal hematopoiesis and maintains HSC symmetric commitment and identity by controlling *Myc* abundance in differentiating HSCs and *Myc* mRNA stability (55). *Metll3* ablation can impair the differentiation of myeloid, megakaryocytes, and erythroid lineages, leading to an additional population that molecularly and functionally resembles multipotent progenitors (55). m^6^A loss by deleting *Metll3* in mice blocked HSC transition to myeloid progenitors, notably presenting as decreases in common myeloid progenitors (CMPs) and granulocyte myeloid progenitors (GMPs) (55). Meanwhile, they also found a cell-intrinsic role of *Metll3* in regulating HSC number and function in a bone marrow competitive transplantation trial (55). In our previous study, we discovered that the *METTL3-mettl14* methyltransferase complex plays a vital role in regulating HSC self-renewal in adult mouse bone marrow, and *METTL3* is mainly responsible for HSCs being in a quiescent state in mice (56). After conditional knockout of *METTL3*, Metll14, or both in mice, we found that the depletion of *METTL3* is in charge of expanding phenotypic HSCs in adult mouse bone marrow and promotes the HSC cell cycle by regulating HSC self-renewal genes such as *Nr4a2*, *p21*, *Bmi-1*, and *Prdm16* (56). In human HSPCs from cord blood, the depletion of *METTL3* has been discovered to enhance cell differentiation, inhibit cell proliferation with fewer colony-forming units (CFUs) in all lineages, and hardly affect the apoptosis of HSPCs (31). Additionally, *METTL3* absence contributes to myeloid differentiation, and *METTL3* mRNA is expressed at lower levels in mature differentiated myeloid cells in both mouse HSCs and HSPCs (31). Taking human erythroleukemia (HEL) cells as a surrogate model for studying erythropoiesis, Kupper et al. also found that *METTL3* blocked erythropoiesis by impacting the stage-specific gene expression of erythroid progenitors, such as the erythroid transcription factors GYPA, HBA1, SPTB, EPOR, and ALAS2 (57).

As *METTL3* plays a significant role in normal hematopoiesis, an increasing number of studies on hematology malignancies have been reported in recent years, including AML, acute lymphocytic leukemia (ALL), CML, and lymphomas.

### Acute Myeloid Leukemia

AML is a common hematological malignancy that is mainly caused by gene mutations and chromosomal aberrations resulting in changes in gene expression and, sequentially, aberrant growth and differentiation of hematopoietic stem cells (HSCs) (65). In 2017, Ly Vu et al. discovered that *METTL3* has higher expression in AML cells than in normal HSPCs (31). In addition, they found that *METTL3* disruption promotes the differentiation and apoptosis of AML cells both *in vitro* (MOLM-13 cells) and *in vivo*, which indicated that *METTL3* affects the undifferentiated state and growth of leukemia cell lines (31). By connecting the single-nucleotide-resolution mapping of m^6^A and ribosome profiling, they revealed that MELLT3 deletion reduced the translation efficiency of *c-MYC*, *BCL2*, and *PTEN* in MOLM-13, resulting in phosphatidylinositol 3-kinase-AKT (PI3K/AKT) pathway activation (31). Meanwhile, Isaia Barbieri reported that *METTL3* is necessary for leukemia cell growth and in maintaining an undifferentiated state (32). They further found that *METTL3* could be recruited by *CEBPZ* to promoters, which led to m^6^A methylation of the respective mRNAs and increased translation. Among the promoters, translation of SP1 was significantly reduced after deleting *METTL3* (32). Wang et al. also reported that *METTL3* was more highly expressed in immature cells than in mature monocytes, and its depletion significantly inhibited cell proliferation and decreased MYC expression and m^6^A levels on *MYC* mRNA (66). Recently, a group of researchers reported that *METTL3* plays a role in inhibiting adipogenesis of bone marrow mesenchymal stem cells (MSCs) and blocking the chemoresistance of acute myeloid leukemia cells by regulating the PI3K/AKT signaling pathway (58). However, *METTL3* expression was significantly decreased in AML-MSCs, which enhanced the adipogenesis and chemoresistance of AML cells (58). They found that *METTL3* impacted the m^6^A modification of AKT1 mRNA, resulting in a decrease in the protein level of AKT1 and an increase in adipogenesis. Correspondingly, activation of the PI3K/AKT signaling pathway contributes to adipogenesis and AML chemoresistance in MSCs (58). In our recent study, we found that *METTL3* is highly expressed in AML patients, which results in poorer prognosis than in AML patients without *METTL3* expression (p=0.017). Furthermore, knockdown of *METTL3* in AML cells (K562 and Kasumi-1) inhibited proliferation and increased apoptosis and differentiation by regulating the p53 signaling pathway. *METTL3* deletion led to decreased *MDM2* expression and MDM2 mRNA transcript stability, which activated the p53 signaling pathway (59).

### Chronic Myeloid Leukemia

CML is caused by the oncogenic BCR-ABL1 fusion gene, which is mainly treated with tyrosine kinase inhibitors (TKIs) (67). However, TKI resistance is still a challenge for CML patients and increases the risk of transfer to AML (68). In 2018, Zaira Ianniello et al. discovered that *METTL3* is a novel oncogene in CML and potentially a therapeutic target for TKI-resistant CML. The m6A methyltransferase complex *METTL3*/*METTL14* and *METTL3* is upregulated in primary CML patients, and its downregulation significantly impairs the proliferation of both primary CML cells and TKI-sensitive and TKI-resistant CML cells (60). Silencing *METTL3* in K562 cells and the TKI imatinib mesylate-resistant K562 cell line (K562r), they found that *METTL3* affects the growth and viability of CML cells directly and indirectly. MYC, as a transcriptional activator, is notably affected by *METTL3* in CML cells, including the protein, mRNA, and premRNA levels. *METTL3* knockdown strongly reduced MYC expression at multiple levels in CML, which consequently regulated the genes associated with RNA metabolism (60). Moreover, they found that the PES1 protein was potentially involved in blocking the cell cycle in G1 phase after METTL3 knockdown in CML cells (60). They showed that *METTL3* both regulates PES1 by methyltransferase activity in the nucleus and directly promotes PES1 translation in the cytoplasm independently of its catalytic activity (60). Lai et al. recently reported that LINC00470 and *METTL3* played a role in chemoresistance and autophagy in CML by regulating phosphatase and tensin homologue (*PTEN*) (61). PTEN, a well-known tumor suppressor, suppresses the activation of PI3K/AKT signaling and subsequently inhibits AKT activity and its downstream pathways (69). In the study, they disclosed that *PTEN* expression was obviously lower in chemoresistant CML cells than in K562 parental cells, which was negatively associated with LINC00470 and *METTL3* (61). More interestingly, they found that overexpression of LINC00470 shortened the half-life of *PTEN* mRNA and enhanced the binding of *METTL3* to PTEN mRNA (61). The depletion of *METTL3* in K562 cells reversed the downregulation and degradation of *PTEN* mRNA and protein induced by LINC00470 and recovered the normal level of m^6^A modification in *PTEN* (61). Accordingly, overexpression of *METTL3*/LINC00470 promoted chemoresistance and reduced autophagy in CML cells by regulating PTEN stability and activating AKT. Fang-Yi Yao et al. reported that *METTL3* was downregulated in CML cells, resulting in a decrease in the protein level of nuclear enriched abundant transcript 1 (*NEAT1*) (70). Furthermore, *METTL3* downregulation in CMLs reduced its ability to modify *NETA1* m^6^A, subsequently enhancing CML cell viability and inhibiting CML cell apoptosis. NEAT1, a lncRNA, is crucial for composing the subnuclear structure paraspeckle and is associated with the progression of hematological malignancies (62).

### Lymphocytic Neoplasm

Lymphocytic neoplasm comprises lymphoblastic leukemia and lymphoma. Similar to AML, acute lymphoblastic leukemia (ALL) is also a severe hematology malignancy and is the most common form of cancer in children (71). However, studies on m^6^A modification in ALL are significantly fewer than those in AML. Congcong Sun et al. reported that the expression of *METTL3* was lower in children with ETV6-RUNX1-positive ALL (63). Meanwhile, they also found that the *METTL3* expression level was reduced in ALL relapse patients compared with non-relapse patients (63). However, they did not find any correlation between *METTL3* expression and some basic clinical characteristics, including age, sex, initial white blood cell count, blast percentage, and LDH level (63). In 2021, a five-center case–control study reported that METLL3 gene polymorphisms were strongly associated with pediatric ALL, mainly including rs1263801 C>G, rs1139130 A>G, and rs1061027 A>C polymorphisms (33). All three polymorphisms were reported to remarkably increase the risk of common B-type and MLL fusion-type ALL in southern Chinese children (33). Additionally, all three polymorphisms were also related to primitive/naive lymphocytes and MRD after chemotherapy. The study showed that patients carrying rs1263801 CC and rs1139130 AA would have a better response to South China Children Leukemia Group chemotherapeutics (SCCLG) chemotherapeutics, and Chinese Children Cancer Group Chemotherapeutics (CCCG) chemotherapeutics are more efficient for patients with rs1061027 (33).

Lymphoma is a well-known hematological neoplasm that mainly includes Hodgkin and non-Hodgkin lymphomas. Diffuse large B-cell lymphoma (DLBCL) is the most common neoplasm in non-Hodgkin lymphoma, which is an aggressive lymphoma with a median survival of <1 year in untreated patients (72). A study regarding the correlation of m^6^A modifications with DLBCL reported that the expression level of *METTL3* is higher in DLBCL tissues/cell lines than in normal lymph nodes/cells (29). Additionally, higher expression of *METTL3* facilitates the proliferation of DLBCL cell lines and viability by regulating the m^6^A mRNA modification of pigment epithelium-derived factor (PEDF), which was usually regarded as inhibitor of canonical Wnt signaling in previous studies (73, 74). Subsequently, they found that overexpression of PEDF can disable the inhibitory effects of *METTL3* silencing on DLBCL cell proliferation (29). These results suggest that the *METTL3*/PEDF axis may have therapeutic potential for DLBCL, but more specific studies are needed for verification.

## The Potential Application in Cancer Therapy

*METTL3* expression is significantly different in different tumors. The above discussion indicates that *METTL3* plays a vital role in both normal hematopoiesis and hematological malignancies, including their self-renewal and differentiation. *METTL3* knockdown can destroy HSPC differentiation and HSC symmetric commitment by regulating *Myc* and *Notch1a* m^6^A modification (14, 54, 55). Despite the discrepancy in *METTL3* expression levels in different hematological malignancies, *METTL3* is upregulated in most tumor tissues and cell lines and is involved in disease progression and the maintenance of a cancer cell undifferentiated state. Higher expression of *METTL3* in AML is critical to maintain the undifferentiation of AML cells, promote the growth of AML cells, and inhibit AML cell apoptosis (31, 32, 66). In CML, TKI resistance always makes it more difficult to cure patients with CML, while *METTL3* has been found to affect the apoptosis, proliferation, and viability of CML cells with higher expression (60, 61, 68, 70). More importantly, *METTL3* depletion notably damages the proliferation of primary CML cells and TKI-resistant CML cells, which suggests that *METTL3* inhibitors may have novel potential to cure TKI-resistant CML patients. Similarly, the expression level of *METTL3* in DLBCL tissues and cell lines is higher than that in normal lymph nodes and cells, which promotes the proliferation of DLBCL cell lines and viability by governing PEDF (29). In contrast, *METTL3* has also been discovered to be downregulated in CML, and it decreases NEAT1 m^6^A modification to impact CML viability and apoptosis (62). Likewise, ALL children with ETV6-RUNX1 positivity had lower METTL3 expression than normal children.

Due to the further understanding of m^6^A modification, associated molecular inhibitors have been produced and studied, such as the *FTO* and *METTL3* molecules. Molecular inhibitors of *FTO*, including meclofenamic acid (MA), MA2, FB23-2, and FB23, have been produced and studied (75–77). Among them, FB23-2 inhibits growth and promotes the differentiation/apoptosis of AML cells both *in vitro* and *in vivo* [patient-derived xenograft (PDX) model] (77). Similar to molecular inhibitors of *FTO*, *METTL3* molecule inhibitors have also been produced and studied *in vitro* and *vivo*, especially in hematology malignances. STM2457 is a highly potent inhibitor of *METTL3* with an IC50 of 16.9 nM, and it performs a cofactor competitive mode using SAM in surface plasmon resonance, avoiding the homocysteine binding pocket used by SAM (78). STM2457 has been verified to block both human AML cell lines (MOLM-13) and proliferation and colony-forming ability potential and promote differentiation and apoptosis, whereas it has no impact on normal hematopoiesis (78). Furthermore, STM2457 application in AML cells significantly reduced the m^6^A modification of several mRNAs associated with AML. Among these mRNAs, SP1 and BRD4, which are known to be governed by *METTL3*, were obviously decreased upon treating MOLM-13 cells with STM2457 (78). Consistent with the *in vitro* results, the inhibition of *METTL3* function by STM2457 was also verified *in vivo*. STM2457 can prevent AML expansion and impair leukemia stem cells in both a patient-derived xenograft (PDX) model and a primary mouse MLL-AF9/Flt3^itd/+^ model (78). Another *METTL3*-selective inhibitor, UZH1a, a high-nanomolar inhibitor, occupies the SAM binding site of *METTL3*, similar to STM2457 (79). UZH1a could also result in a decrease in the mRNA m^6^A methylation level in AML MOLM-13 cells in a dose-dependent manner (IC50 of 7 µM) (79). Furthermore, this group also confirmed that UZH1a could reduce mRNA m^6^A/A levels not only in the leukemia cell line MOLM-13 but also in other cell lines (osteosarcoma U2OS cells and immortalized human embryonic kidney cell line HEK293T).

## Conclusion

Increasing studies have identified that m^6^A RNA modifications notably influence physiological and pathological processes in eukaryotes by regulating RNA translation, degradation, stability, export, and splicing. Meanwhile, many recent emerging studies have revealed that m^6^A RNA modifications play critical roles in various cancers, including cervical cancer, hepatocellular carcinoma, leukemia, lymphoma, glioblastoma, lung cancer, nasopharyngeal carcinoma, and bladder cancer. Therefore, more attention should be given to the function of m^6^A modification in tumorigenesis, which would provide more suitable therapies for patients.

In the reversible and dynamic m^6^A process, *METTL3*, with a special structure, is the core methyltransferase involved in m^6^A modification. It plays a vital role in many biological processes, including cell differentiation, proliferation, viability, apoptosis, cycle, invasion, inflammatory response, and metabolism (80). Moreover, it has also been reported that *METTL3* in the cytoplasm can promote translation independently of its methyltransferase activity (51). Meanwhile, recent studies have revealed that *METTL3* impacts biological processes in both normal and malignant hematopoiesis. *METTL3* not only influences normal and symmetric HSPC/HSC differentiation, HSPC self-renewal, and colony formation ability in normal hematopoiesis but also affects leukemia cell differentiation, proliferation, apoptosis, chemoresistance, and a higher risk of specific ALL or lymphoma. Therefore, the initial mechanisms of *METTL3* in hematological biology and disease require further exploration, subsequently revealing the relationship between them and providing a foundation for producing potential inhibitors. However, the role of other members of the m6A process, such as methyltransferases and demethyltransferases, should also be considered in tumorigenesis. Undoubtedly, the deeper the understanding of m^6^A modification, the more inhibitors will be produced. Similar to *FTO* inhibitors, *METTL3* inhibitors have been produced and studied in recent years, and they will be a potent potential target to treat patients with hematological malignancies in the future, especially AML and chemoresistant CML.

Collectively, *METTL3* plays a vital role in both normal and malignant hematopoiesis, while its studies are still in a very early stage. Therefore, further studies are required to explore the mechanism, hoping to optimize a potential targeted *METTL3* therapy and use it widely in clinical practice in the future.

## Author Contributions

XW and YG wrote and revised the manuscript. WY drafted the pictures. All authors contributed to the article and approved the submitted version.

## Funding

The study was supported by the Foundation of the Science and Technology Department of Sichuan Province (No. 2019YFS0026).

## Conflict of Interest

The authors declare that the research was conducted in the absence of any commercial or financial relationships that could be construed as a potential conflict of interest.

## Publisher’s Note

All claims expressed in this article are solely those of the authors and do not necessarily represent those of their affiliated organizations, or those of the publisher, the editors and the reviewers. Any product that may be evaluated in this article, or claim that may be made by its manufacturer, is not guaranteed or endorsed by the publisher.
